# Trends of genetic contributions on epigenetic clocks and related methylation sites with aging: A population‐based adult twin study

**DOI:** 10.1111/acel.14403

**Published:** 2024-11-14

**Authors:** Xuanming Hong, Hui Cao, Weihua Cao, Jun Lv, Canqing Yu, Tao Huang, Dianjianyi Sun, Chunxiao Liao, Yuanjie Pang, Runhua Hu, Ruqin Gao, Min Yu, Jinyi Zhou, Xianping Wu, Yu Liu, Shengli Yin, Wenjing Gao, Liming Li

**Affiliations:** ^1^ Department of Epidemiology and Biostatistics, School of Public Health Peking University Beijing China; ^2^ Key Laboratory of Epidemiology of Major Diseases, Ministry of Education Peking University Beijing China; ^3^ Qingdao Center for Disease Control and Prevention Qingdao China; ^4^ Zhejiang Center for Disease Control and Prevention Hangzhou China; ^5^ Jiangsu Center for Disease Control and Prevention Nanjing China; ^6^ Sichuan Center for Disease Control and Prevention Chengdu China; ^7^ Heilongjiang Center for Disease Control and Prevention Harbin China; ^8^ Dezhou Center for Disease Control and Prevention Dezhou China

**Keywords:** aging, epigenetic clock, longitudinal studies, twin study

## Abstract

Several crucial acceleration periods exist during aging process. Epigenetic clocks, serving as indicators of aging, are influenced by genetic factors. Investigating how the genetic contributions on these clocks change with age may provide novel insights into the aging process. In this study, based on 1084 adult twins from the Chinese National Twin Registry (CNTR), we established structural equation models (SEMs) to evaluate the trends in genetic influence with aging for epigenetic clocks, which include PC‐Horvath, PC‐Hannum, PC‐PhenoAge, PC‐GrimAge, and DunedinPACE. A decline in overall heritability was observed for all five clocks from ages 31 to 70, with a relatively stable trend at first. Subsequently, apart from PC‐GrimAge, the other four clocks displayed a more evident drop in heritability: DunedinPACE and PC‐PhenoAge experienced a clear decline between 55 and 65 years, while PC‐Horvath and PC‐Hannum showed a similar decrease between 60 and 70 years. In contrast, the heritability of PC‐GrimAge remained stable throughout. An analysis of methylation sites (CpGs) from these clocks identified 41, 26, 4, and 36 CpG sites potentially underlying heritability changes in DunedinPACE, PC‐Horvath, PC‐Hannum, and PC‐PhenoAge, respectively. Data from the CNTR were collected through two surveys in 2013 and 2018. Based on 308 twins with longitudinal data, declines in genetic components were observed at follow‐up compared to baseline, with significant decreases in the four PC‐clocks. DunedinPACE peaked in 5‐year longitudinal genetic contribution changes at age 55–60, while PC‐clocks consistently peaked at age 50–55. These findings may offer novel insights into the role of genetic variations in aging.

## Graphical abstract

The genetic contributions to epigenetic clocks demonstrated an overall decline between ages 31 and 70. Heritability remained relatively stable from ages 31 to 55, followed by a more pronounced decrease from ages 55 to 70 for most of the clocks, with the steepest decline observed between ages 60 and 64.

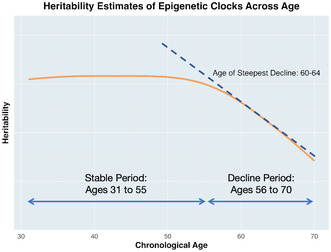

**Abbreviations**
CNTR: Chinese National Twin Registry
CpG: 5′‐cytosine‐phosphate‐guanine‐3′
DNAm: DNA methylation
DTW: dynamic time warping
DZ: Dizygotic twins
FDR: false discovery rate
LOSEM: local structural equation modeling
MZ: monozygotic twins
PC: principal component
SD: standard deviation
SEMs: structural equation models
SNPs: single‐nucleotide polymorphisms

## 1 INTRODUCTION

Aging is a primary risk factor for the majority of chronic diseases, with the aging of the global population, the incidence of senescence‐related disorders is increasing (Robinson et al., 2018). The pace of population aging represents an important healthcare and social issue, thus, to understand the molecular basis of aging was essential and could be instrumental in identifying possible approaches for therapeutic intervention (Maeso‐Diaz et al., 2018; Sun et al., 2015). During the aging process, particular periods that aging pace spikes may exist, accompanied by variations in physiological functions and molecular mechanisms (Han, 2024). A typical example is that menopause may signify a critical period that has been shown to accelerate aging (Levine et al., 2016). A study depicted waves of changes in distinct biological pathways across different age groups and discovered that, around the time of menopause, women may experience a peak change at the levels of phenomics, transcriptomics, proteomics, and metabolomics (Li et al., 2023). By analyzing plasma proteins in participants aged 18 to 95, another study identified three peaks of plasma protein level changes at the ages of 34, 60, and 78, suggesting that these ages may be crucial change points in the aging process (Lehallier et al., 2019).

DNA methylation (DNAm), as a heritable and stable epigenetic alteration, is considered to play an essential role in cellular senescence and aging (Lleonart et al., 2009). Epigenetic clocks based on DNAm has emerged as a widely used biomarker of aging, surpasses various biomarkers of healthy aging in its accuracy and utility (Fiorito et al., 2021). Several epigenetic clocks have been developed based on different criteria, each reflecting various aspects of the aging process. The first‐generation clocks, most notably the Horvath and Hannum clocks, were established using chronological age as the training phenotype, were highly correlated with chronological age, with a correlation coefficient as high as 0.96 (Hannum et al., 2013; Horvath, 2013). The second‐generation clocks, represented by the PhenoAge and GrimAge clock, incorporate mortality risk and related biomarkers into the training phenotype, demonstrating a pronounced advantage in predicting mortality (Levine et al., 2018; Lu et al., 2019). The third‐generation clocks (represented by DunedinPACE) are constructed based on longitudinal changes in multiple biomarkers which can measure the pace of aging (Belsky et al., 2020).

Aging has long thought to be a genetically driven process, and epigenetic clocks were also reported to be influenced by genetic factors (Lu et al., 2018; Xie et al., 2022). The impact of genetic factors on methylation has demonstrated to be varied with increasing age throughout the lifecycle (Hannum et al., 2013; Reynolds et al., 2020; Shah et al., 2014). Thus, understanding the changing influence of genetic components in epigenetic clocks can provide insights for comprehending critical period of aging and potentially modulating the process of senescence.

Twins provide a valuable research tool for studying epigenetic changes and the underlying genetic contributions. The genetic background was shared within twin pairs, which was 50% on average of segregating genes for dizygotic (DZ) twins and 100% for monozygotic (MZ) twins (Dempster et al., 2011). Utilizing twin study designs allows us to estimate the extent of genetic influence on epigenetic clocks. Several twin research has quantitatively assessed the genetic effects of epigenetic clocks. A study that included 31 female twin pairs has estimated the Horvath clock heritability at approximately 40%, while another study based on 104 twin pairs revealed a heritability of 55% at baseline (age 69) and 51% at follow‐up (age 79) for Horvath clock (Horvath, 2013; Jylhava et al., 2019). Although a longitudinal follow‐up study in late life has been conducted, to our knowledge, no prior study has investigated the trajectory of genetic contributions to epigenetic clocks across a broader range of ages. Further investigation of the sources of genetic variation across different age groups in a longitudinal context could provide novel insights into genetically driven aging‐associated changes, identify pathways of plasticity, and elucidate the biological mechanisms underlying temporal variability (Jylhava et al., 2019; Reynolds et al., 2020).

Based on the twin data from the Chinese National Twin Registry (CNTR), the aims of this study were to:

①Assess the changes in heritability of epigenetic clocks and their CpGs with aging, identify critical times where significant changes in heritability occur;

②Identify possible CpGs that responsible for the change trends in heritability of epigenetic clocks with aging, and enrich these CpGs to uncover the specific biological functions and related pathways;

③Utilize longitudinal data to examine the changes of genetic components in epigenetic clocks, and further explore and validate crucial times with fluctuations of genetic contributions during the course of human aging.

## 2 METHODS

### 2.1 Study population and zygosity assessment

Data analyzed in this study were from the CNTR, which conducted surveys in both 2013 and 2018, including questionnaire inquiries, physical examinations, and blood withdrawal. A detailed description of the CNTR's study design, methods for data collection, and the characteristics of the study population has been previously documented (Gao et al., 2019). The questionnaire inquiries encompassed an extensive range of topics, including demographic information, medical backgrounds, and lifestyle factors. Genome‐wide DNA methylation (DNAm) profiling was conducted using whole blood samples. The research protocol of this study was subject to ethical approval by the Biomedical Ethics Committee at Peking University, Beijing, China. All participants involved in the study provided written informed consent (reference number: IRB00001052‐22032, IRB00001052‐13022, IRB00001052‐14021).

Eligibility for participation in this study including the following criteria: (1) completion of questionnaires and provided comprehensive details on demographics for each member of the twin pairs; (2) blood withdrawal has completed from both individuals in the twin pairs; (3) participation in at least one survey in 2013 or 2018. Pregnant individuals and their cotwins were excluded from the study. Additionally, if one member of a twin pair was removed in subsequent steps of DNA methylation (DNAm) data quality control or analysis procedures, his/her cotwin will also be excluded. The initial study population encompassed 1088 subjects (average age = 49.9 ± 12.2 years). Among these, longitudinal data were obtainable for 314 subjects (representing 28.8% of the total study cohort), who had completed both surveys in 2013 and 2018.

The determination of twin zygosity was carried out through a panel comprising 59 single‐nucleotide polymorphisms (SNPs) on the Illumina Infinium Methylation Chips. Twins with concordance greater than 90% in these SNPs were classified as monozygotic (MZ) twins (Wang et al., 2015). 816 twins (196 twins with longitudinal data) were classified to be MZ in this study.

### 2.2 DNA methylation assessment

DNA were extracted from peripheral blood and subjected to bisulfite conversion using the EZ DNA Methylation Kit (Zymo Research, Orange County, CA, USA). Subsequently, the whole‐genome DNA methylation profile was quantified with the use of either the Illumina Infinium Human Methylation 450 K or EPIC BeadChip (Illumina, San Diego, CA, USA). The 450 K array was employed on 326 and 123 samples from the 2013 survey for cross‐sectional and longitudinal analyses, respectively. The EPIC BeadChip was used for cross‐sectional and longitudinal analyses on 762 and 314 samples, respectively, from the 2018 survey, as well as for longitudinal analysis of 191 samples from the 2013 survey. Probes from both BeadChip types were included in the analysis. The reproducibility of assays between the two BeadChips for the samples was previously assessed and found to be 98%. Additionally, it was demonstrated that 90% of the probes on the 450 K array were reproducibly detectable using the EPIC BeadChip (Pidsley et al., 2016). Data from both the Illumina EPIC and 450 K platforms were merged through the application of the “combineArrays” function within the R package “minfi” (version 1.34.0), resulting in a combined dataset (Aryee et al., 2014).

The methylation levels at each CpG site were expressed as β‐values, which denote the average methylation level ranging from 0 (no methylation) to 1 (complete methylation). The *β*‐value was calculated through the equation: *β* = *M*/(*M* + *U* + 100), where *M* and *U* stands for the mean signal intensity of methylated probes and unmethylated probes for each site. The assessment of M and U was performed using the R package “minfi” (Aryee et al., 2014). Next, β‐values underwent a process of quantile normalization. To further refine the data, adjustments for blood cell counts were implied using the “ChAMP” package in R (version 2.18.3) (Tian et al., 2017). In order to minimize confounding effects from the DNAm detection procedures, DNAm data across assays were adjusted for batch variations using the ComBat method provided by the “sva” R package (version 3.38.0) (Leek et al., 2012).

Several quality control protocols were implemented on the DNAm data to filter out probes and samples below quality requirements. The protocols include: (1)Probes that did not display significant differences (*p* > 0.01) when comparing the signals from the CpG sites to those of blank controls; (2)Probes with a minor allele frequency (MAF) >0.01 or were annotated with single nucleotide polymorphisms (SNPs) on the microarray platform; (3) missing probes or had a detection *p*‐value >0.01 in over 1% of the samples; (4) Cross‐reactive probes; and (5) Samples demonstrating missing rates of more than 1% in probes.

For our analysis, the initial DNAm dataset included a total of 378,654 CpGs that were present on both the 450 k and EPIC arrays and 1084 participants for subsequent analysis procedures. Of these, a subset consisting of 308 subjects provided longitudinal DNAm data.

### 2.3 Epigenetic clocks calculation

The epigenetic clocks analyzed in this study include DunedinPACE and principal component (PC) based clocks. DunedinPACE denotes the pace of aging. A DunedinPACE value exceeding 1 denotes an accelerated aging state, whereas a value below 1 suggests a deceleration of aging (Belsky et al., 2022). The calculation of DunedinPACE was conducted by the “DunedinPACE” package in R.

PC‐based clocks compute principal components from the CpG sites derived from foundational clocks, consisting of Horvath, Hannum, PhenoAge, GrimAge clocks. These principal components served as the input variables for the prediction of biological age, and then employed to retrain the PC versions of these clocks. The PC‐based clocks have been assessed to obsess high reliability and may be instrumental in personalized medicine, longitudinal tracking, in vitro studies and clinical trials of aging interventions (Higgins‐Chen et al., 2022). PC‐clocks were estimated utilizing the R code provided by the researchers (https://github.com/MorganLevineLab/PC‐Clocks/).

### 2.4 Statistical analysis

**FIGURE 1:**
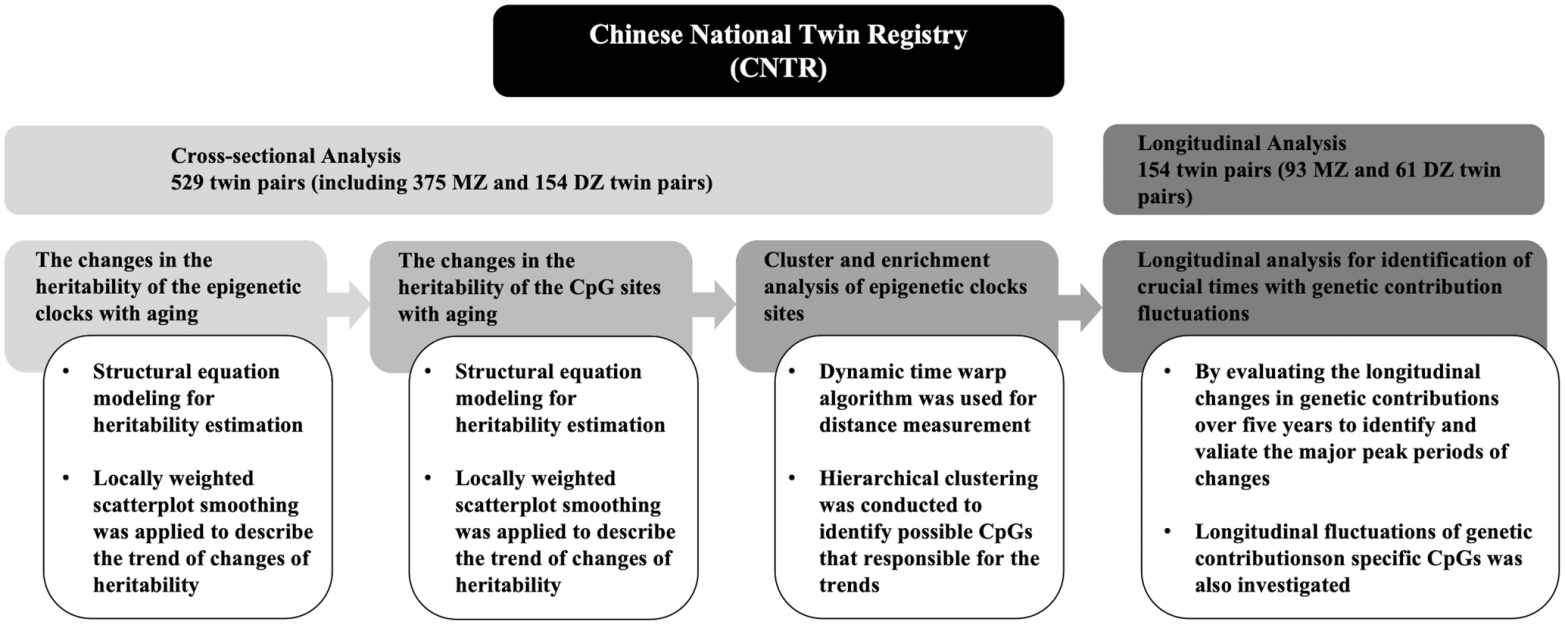
Schematic of study design. CpG indicates 5′‐cytosine‐phosphate‐guanine‐3′ in DNA; MZ, monozygotic twins; DZ dizygotic twins.

The overall study design is summarized in Figure 1. Analyses were all conducted using R software (R statistics), version 4.3.1.

### 2.5 Heritability analysis of epigenetic clocks and their CpGs

First, the R package OpenMx (version 2.21.8) was employed to evaluate the relative genetic and environmental contributions to the variance for each of the epigenetic clocks in the general study population (Neale et al., 2016). Then, we assessed the age‐related changes in the genetic contributions of each epigenetic clock and their included CpG sites using the “umxGxE_window” function from the R package umx (version 4.20.0). This function applies local structural equation modeling (LOSEM) to estimate how the genetic contribution of one variable varies across different levels of another variable by using a moving‐window approach (Bates et al., 2019). Both methods employed structural equation models (SEMs).

The SEMs utilized in the twin study are documented in previous literature (Huang et al., 2018). In summary, by using the different similarity between MZ twins (shared 100% of their genetic information) and DZ twins (share an average of 50% of their genes), the variation (i.e., variance) in phenotype was decomposed into four components: ① A, representing additive genetic variance; ② D, indicating nonadditive genetic variance; ③ C, the common environmental variance shared among twins; and ④ E, the unique environmental variance attributable to nonshared environmental influences within twins. A fully saturated model that represent the accurate mean, variance, and covariance structures of the observed dataset was established first. Subsequently, sub‐models that constraining different components to zero will be employed to evaluate whether there was a better more parsimonious fit to the data. Given the previous twin studies that focused on epigenetic clocks and DNA methylation data have predominantly determined the AE model (model consisting of A and E components) to be the most optimal and parsimonious submodel, the current study similarly adopted the AE model for fitting the SEMs to ensure comparability and interpretability of outcomes (Jylhava et al., 2019; Reynolds et al., 2020).

Figure S1 exemplifies the decomposition of variance in the AE model. Since monozygotic (MZ) twins share 100% of their genetic composition, the difference between the twin pairs was primarily attributable to the unique environmental variance (E). Therefore, the correlation of MZ twins serves as an estimator for the effects of additive genetic variance A. On the other hand, correlation of DZ twins provides an estimate of ½A. Based on the above estimations, the heritability is inferred as a proportion of relative genetic contributions (A) (Berry et al., 2020). Age and sex were included as covariates in the structural equation models (SEMs) constructed using the R package OpenMx. In contrast, age was used as a moderator in the LOSEMs constructed with the R package umx to examine the genetic contributions of these epigenetic biomarkers across an age range of 30 to 70 years.

### 2.6 Cluster and pathway enrichment analysis of methylation clocks sites

With the exception of PC‐GrimAge, a clustering analysis was conducted on the trajectories of changes in the heritability of the epigenetic clocks along with their corresponding methylation sites with aging. The main objective of this analysis was to elucidate potential causative specific CpGs/genes that may drive the genetic variations observed in the heritability of the epigenetic clock.

To capture the characteristics of the trajectories that represent changes in the heritability of these phenotypes with age, we employed the Dynamic Time Warping (DTW) algorithm (Zheng et al., 2016). DTW is a dynamic programming algorithm that assures the identification of the optimal alignment between sequences of values (Loose et al., 2016). The DTW algorithm was applied using the R package “dtw” (version 1.23–1) and its function “dtwDist.”

Then, the estimation of optimal numbers of clusters was conducted via the gap statistic method, by untilizing “fviz_nbclust” function from the “factoextra” package (version 1.0.7). Based on the optimal clustering numbers, the clustering analysis were performed using hierarchical clustering with the Ward method by the “hclust” and “cutree” function from the “stats” package (version 4.3.1) (Tibshirani et al., 2001; Ward, 1963).

Subsequently, pathway enrichment analyses were conducted utilizing the “enrichGO” function in the “clusterProfiler” package (version 4.8.1). Human gene annotation was carried out employing the “org.Hs.eg.db” package (version 3.17.0). Pathways achieving a False Discovery Rate (FDR) adjusted *p*‐value <0.05 were considered as statistically significant.

### 2.7 Longitudinal study for genetic components of epigenetic clocks and their CpGs with aging

Based on the twins with longitudinal DNAm data, we further conducted a longitudinal analysis on the changes in the genetic components of epigenetic clocks and their CpGs, to further explore the critical time periods at which the genetic influences of the epigenetic clocks changes. The values of genetic variance components instead of heritability were used in the longitudinal analyses, as heritability is a proportion, whereas variance components are more preferable for comparisons among variables in different time periods. The univariate SEMs that detailed above were also modeled to estimate the 5‐year changes in relative contributions to clocks and specific CpGs. Due to the limited sample size of the longitudinal data, we grouped the study population into 5‐year age groups starting with ages 30 at baseline and ending with 65 at baseline (corresponding to ages 35 and 70 at follow‐up) to calculate the longitudinal changes with aging. We then estimated the effects of genetic components separately for the full study population and for each 5‐year age groups.

Firstly, the genetic contributions (component A) of epigenetic clocks and their associated sites for full study subjects and for each age group were evaluated based on data from the years 2013 and 2018, respectively. Then, the alteration in genetic components across a 5‐year longitudinal span for methylation clocks and their corresponding CpG sites was computed by subtracting the components at baseline from the components from corresponding groups at follow‐up (i.e. 5‐years later for the full population, the next age group 5‐years later for each age group). Within this analysis, the selection of models, the adjustment of covariates, and the evaluation of the model paths were conducted consistent to the heritability analysis that above described.

## 3 RESULTS

Table 1 describes the detailed demographic information of the participants. The 1084 participants included 758 MZ twins, 733 males and had a mean age of 50.0 (SD = 12.1) years old. The means (SD) of methylation clocks were as follows: 1.13(0.11) for DunedinPACE, 61.28(7.50) for PC‐Horvath, 66.64(8.12) for PC‐Hannum, 55.68(10.47) PC‐PhenoAge, and 65.1(10.0) for PC‐GrimAge (Table 1). The correlation coefficients between epigenetic clocks and chronological age varied from 0.38 to 0.96 (Table S1).

**TABLE 1:** Characteristics of the analytic samples by study group.

	Cross‐sectional analysis	Longitudinal analysis		*p*‐values
	Total	Baseline	Follow‐up	
*N*	1084	308		
Age, years	50.0 ± 12.1	50.2 ± 10.2	54.9 ± 10.2	0.71
Female, *n* (%)	341 (31.5)	121 (39.3)		0.02
MZ, *n* (%)	758 (69.9)	186 (60.4)		<0.01
DunedinPACE	1.1 ± 0.1	1.1 ± 0.1	1.1 ± 0.1	0.67
PC‐Horvath	61.3 (7.5)	62.1 ± 6.7	63.6 ± 6.7	0.04
PC‐Hannum	66.6 (8.1)	70.0 ± 6.9	72.8 ± 6.8	<0.01
PC‐PhenoAge	55.7 (10.5)	58.3 ± 8.5	62.6 ± 8.2	<0.01
PC‐GrimAge	65.1 (10.0)	64.9 (8.2)	68.9 (8.2)	0.85

*Note*: *p*‐values were derived from comparisons between the full study population and the baseline population, with continuous variables assessed using two‐sample *t*‐tests and categorical variables evaluated using chi‐square tests.

Abbreviation: MZ, monozygotic twins.

### 3.1 The changes in the heritability of the epigenetic clocks with aging

To begin with, heritability estimates for the DunedinPACE, PC‐based clocks and related indicators were calculated across the entire study population. The heritability of the clocks was all found to be moderate high: DunedinPACE at 0.62, PC‐Horvath at 0.69, PC‐Hannum at 0.72, PC‐PhenoAge at 0.62, and PC‐GrimAge at 0.58 (Table S2).

**FIGURE 2:**
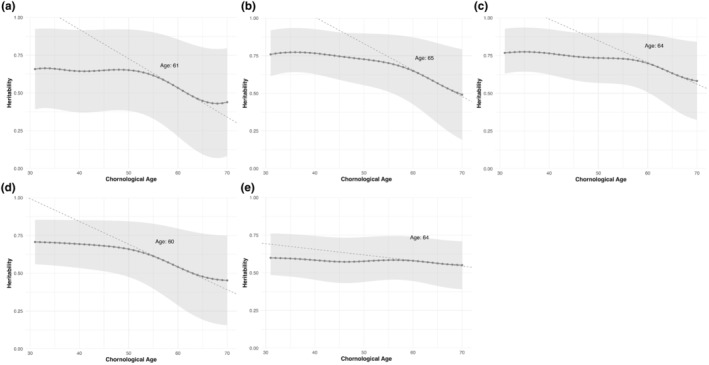
Heritability for epigenetic clocks with aging. Line chart of heritability with aging for (a) DunedinPACE; (b) PC‐Horvath; (c) PC‐Hannum; (d) PC‐PhenoAge; (e) PC‐GrimAge.

Then the changes in heritability of the epigenetic clocks with aging was estimated by applying the LOSEM approach. To improve the fits of univariate SEMs, the heritability analysis of epigenetic clocks was restricted to individuals whose actual ages ranged from 30 to 70 years, due to the relatively limited number of participants below and above this age range (The number of subjects for each age was detailed in Table S3). Figure 2 displays the variation in heritability across five different measures of methylation clocks with aging. For all five methylation clocks, the overall heritability demonstrated a declining trend from chronological age 30 to 70. The point estimates of heritability were highest at 0.66 at age 31 for DunedinPACE and decreased to 0.44 by age 70. PC‐Horvath exhibited the greatest decrease, starting from a peak of 0.77 at age 33 and declining to a minimum of 0.49 by age 70. The point estimates of heritability were highest at 0.77 at age 31 for PC‐Hannum and decreased to its lowest point at 0.58 by age 70. PC‐PhenoAge revealed a maximum heritability of 0.71 at age 31, which decreased to 0.45 at age 70. PC‐GrimAge showed a relatively smaller decline from its highest point estimates of heritability of 0.60 at age 31 to the lowest at 0.55 by age 70 (Table S4).

We used dashed lines to indicate the age points between 31 and 70 years at which the heritability of epigenetic clocks declines most rapidly in the Figure 2. For all clocks, this point occurs between 60 and 64 years. The overall trends for heritability of all the epigenetic clocks firstly follows a relatively flattening trajectory (31–55 years for DunedinPACE and PCPhenoAge; 31–60 years for PCHorvath, PCHannum and PCPhenoAge). This pattern suggests a continuous and stable decrease in genetic influence across these age ranges. However, there is a noticeably declines in heritability near the age points of the most rapid decrease: between the ages of 55 and 65, the point estimates of heritability declines from 0.62 to 0.45 for DunedinPACE, and that of PCPhenoAge decreases from 0.61 to 0.48; between ages 60 and 70, the point estimates of heritability decreases from 0.65 to 0.49 for PC‐Horvath, and that of PC‐hannum drops from 0.70 to 0.58. This suggests that genetic influence may undergo significant changes during these period (Table S4).

### 3.2 Heritability of the methylation clocks sites with aging & cluster and pathway enrichment analysis

DunedinPACE and the original versions of PC‐Horvath, PC‐Hannum, and PC‐PhenoAge clocks (i.e., Horvath, Hannum, PhenoAge clocks) were composite measures of 173, 353, 71, and 513 CpG sites, respectively. Among these, 152/326/60/499 sites were present in the DNAm data from our study. Heritability of the methylation levels at these sites with aging were assessed in this study (Tables S5–S8). Following this, we employed the DTW algorithm to analyze the age‐related trends in the heritability of these sites, through quantitatively calculating the distances between trajectories of each site's heritability to capture their dynamic characteristics (Tables S9–S12).

The trajectories of CpG heritability changes were then clustered along with that of corresponding epigenetic clocks to examine the similarities of these trajectories. The optimal number of clusters was estimated to be 7 for DunedinPACE clocks sites, 3 for PC‐Horvath sites, 4 for PC‐Hannum sites, 6 for PC‐PhenoAge sites, respectively, based on gap statistic method (Figure S2). Then, in the hierarchical cluster analysis based on the determined optimal number of clusters, the trajectories of the four clocks were respectively clustered with 41, 26, 4, and 36 corresponding CpG sites, as presented in Table 2. This indicates that the temporal variations in the heritability of these epigenetic clocks may be influenced by these specific sites. Additionally, to evaluate the significance of the CpGs in the establishment of these clocks, we conducted comparisons between clusters of CpGs based on the coefficients from clocks. Our findings revealed no statistically significant differences between the coefficients of CpGs that clustered with the clocks and those of other sites: the p‐values from *t*‐tests were 0.59 for DunedinPACE, 0.24 for PC‐Horvath, 0.05 for PC‐Hannum, and 0.88 for PC‐PhenoAge. The clustering results and annotation information for the CpG sites are detailed in Tables S13–S16. Enrichment analyses of these CpG sites with potential influence identified five Horvath clock‐associated sites enriched in pathways related to the regulation and positive modulation of cell–cell adhesion, leukocyte cell–cell adhesion, chondrocyte differentiation, and cartilage development, while one site from the Hannum clock was involved in the regulation of synapse and postsynapse organization, as well as the activation and positive regulation of GTPase activity (Table S17).

**TABLE 2:** CpGs exhibiting genetic variation trajectories in close association with epigenetic clocks.

Clocks	Clustered CpGs
DunedinPACE	cg00835193(LINGO3), cg01055871(EHD2), cg02997983, cg04105250(GAD1), cg04305539(ZNF695), cg05085844(ADAP1), cg05239308(TTLL13), cg05487507(LOC404266;HOXB5), cg06500161(ABCG1), cg06570125, cg06797880(HHIPL1), cg06961233, cg07589381(CASZ1), cg08790676, cg09933458, cg10017843(PARD6A;ACD), cg10053507(KIAA1026), cg11095122(CSGALNACT1), cg11202345(LGALS3BP), cg11452501(LY6G5C), cg11787522(STRA6), cg11835347(RHOC), cg13614083(KCNAB2), cg13945148, cg15829969, cg16413763(MYO10), cg16969872(RBM26), cg17061862, cg17749946(HDGFL1), cg17901584(DHCR24), cg18181703(SOCS3), cg18513344(MUC4), cg20964064(KCNQ2), cg21566642, cg22367678, cg22488164(PLBD1), cg24396875(PMS1), cg24737193, cg25243766, cg25368647(MXD3), cg26264318(BRD2)
PC‐Horvath	cg01353448(C7orf16), cg01459453(SELP), cg02489552(CCDC105), cg03760483(ALOX12), cg04452713(DST), cg06738602(PTGER2), cg07455279(NDUFA3), cg07770222(C8orf31), cg09646392(TNFSF13B), cg09809672(EDARADD), cg13302154(MGP), cg16168311(APOA1BP), cg16744741(PRKG2), cg17589341(SLC14A1), cg18440048(ZNF70), cg19724470(CD274), cg20240860(ACCS), cg20761322(CIB2), cg22449114(TCF15), cg22901840(DIRAS3), cg24450312(RASSF5), cg25101936(ZBTB16), cg25809905(ITGA2B), cg26372517(TFAP2E), cg26453588(BIK), cg27202708(C1orf65)
PC‐Hannum	cg09809672(EDARADD), cg10501210, cg22796704(ARHGAP22), cg23744638
PC‐PhenoAge	cg00083937(MAPK8IP2), cg00338702(CHFR), cg00412772(YIF1B; C19orf33), cg00862290(KCNMB3), cg01114088(CYTL1), cg02228185(ASPA), cg06295856(CALCA), cg06327515(PCDHB14), cg07073964, cg07211259(PDCD1LG2), cg08475827(RIF1), cg08586737(GCC1), cg09096950(UBE4A), cg09404633(LMOD1), cg09799873(NDUFA7;RPS28), cg09809672(EDARADD), cg10669058(CILP2), cg10917602(HSD3B7), cg12864235(CDH9), cg14918082(KCNAB3), cg17133388(FAM162A;CCDC58), cg17627559(TSPAN32;C11orf21), cg17749443(ZFP37), cg18881501(MAP2K3), cg19149785(KLK9;KLK8), cg19297232(SMPD3), cg19514469(ELMO3), cg19724470(CD274), cg20761322(CIB2), cg21201109(KANK2), cg21993406(CENPH), cg22580353(PVR), cg22971191(SLC10A2), cg25536676(DHCR24), cg26201213(MGMT)

### 3.3 Longitudinal changes on the genetic contributions of the epigenetic clocks and their sites with aging

To begin with, the longitudinal changes in genetic components for epigenetic clocks and corresponding CpGs were estimated in the full analysis population. For all the five clocks analyzed, the genetic variance components showed a general decrease trend over a 5‐year period: the A component of DunedinPACE decreased from 0.57 at baseline to 0.49 at follow‐up, from 0.25 to 0.22 for PC‐Horvath, from 0.15 to 0.13 for PC‐Hannum, from 0.20 to 0.10 for PC‐PhenoAge, and from 0.06 to 0.04 for PC‐GrimAge (standardized values of clocks were used in these analyses). Subsequently, we conducted homogeneity tests to examine the equality of variance components in epigenetic clocks between the years 2013 and 2018. The values of genetic components of clocks at baseline were assigned to follow‐up in the test, and if the results of homogeneity models showed a poor fit, variance components were suggested to have statistically significant changes over time. A significant reduction in variability was observed for four PC clocks (all *p*‐values <0.01) in the homogeneity tests, whereas the decreasing trend for DunedinPACE was not statistically significant (*p* = 0.89). For the CpGs from these clocks, a decrease in genetic contribution was observed among 80 sites for DunedinPACE, 141 for PC‐Horvath, 20 for PC‐Hannum, and 237 for PC‐PhenoAge. Within these, declining trends at 13, 23, 7, and 28 sites respectively demonstrated statistical significance in homogeneity tests. Conversely, an increase was recorded at 73 sites for DunedinPACE, 182 for PC‐Horvath, 40 for PC‐Hannum, and 261 for PC‐PhenoAge. Of these increases, 9, 26, 5, and 41 sites respectively remained significant in the homogeneity tests (Tables S18–S21).

Next, the role of genetic contributions (A) of five different epigenetic clocks at each age group at baseline (2013) and follow‐up (2018) survey were calculated. Then, the components at each age group at baseline were subtracted from the corresponding age at follow‐up (e.g., 50–55 group at baseline corresponding to 55–60 group at follow‐up) to obtain the 5‐year longitudinal change in the genetic components of these methylation clocks with aging (Table S22).

**FIGURE 3:**
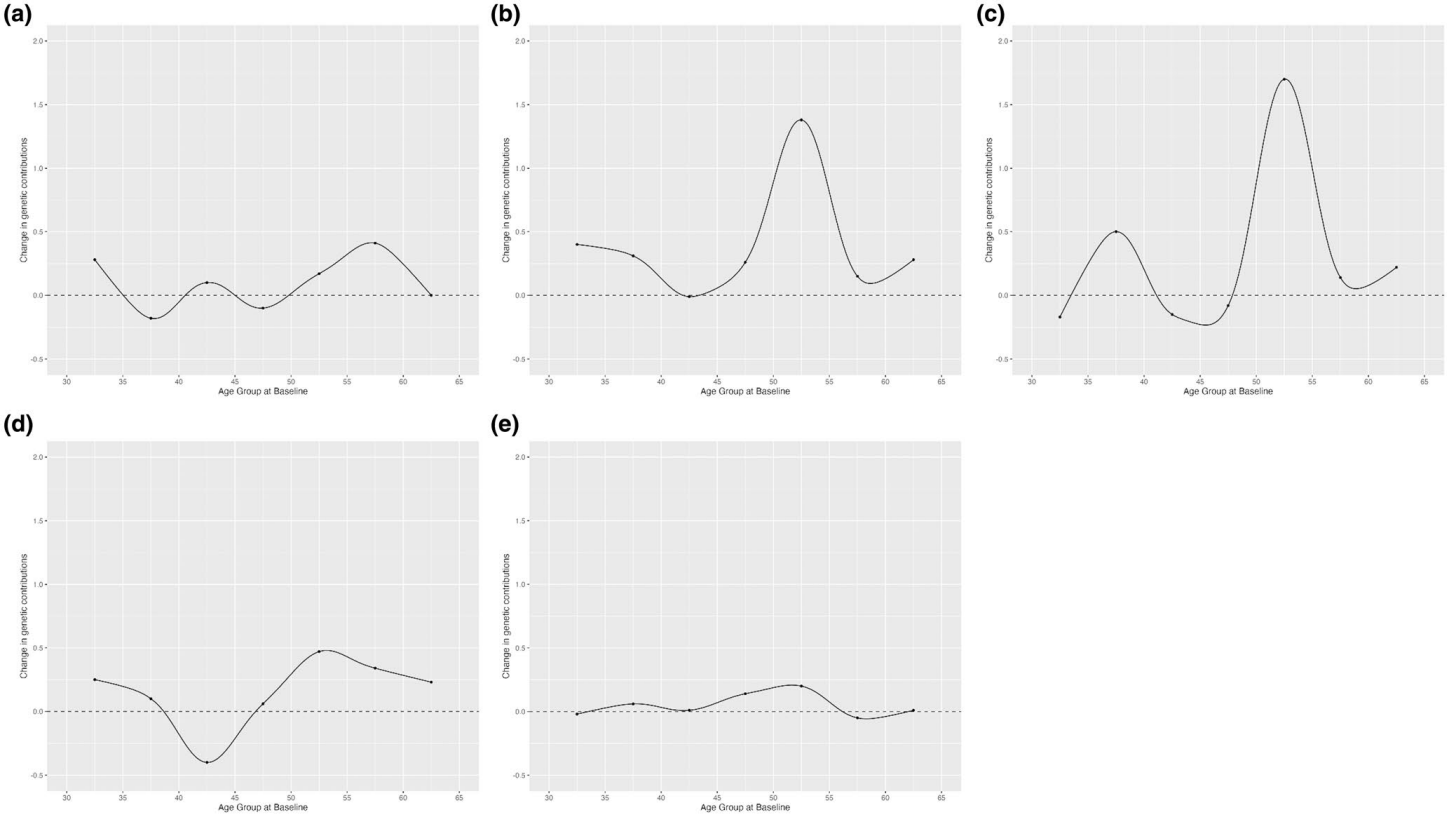
5‐year longitudinal changes in heritability of epigenetic clocks with aging. Line chart of 5‐year longitudinal changes in heritability in each age group for (a) DunedinPACE; (b) PC‐Horvath; (c) PC‐Hannum; (d) PC‐PhenoAge; (e) PC‐GrimAge.

Figure 3 illustrates the trends of 5‐year heritability change of the five different methylation clocks across age groups. DunedinPACE reaches its major peak of change at baseline age group 55–60, suggesting a significant variation in the genetic influence on DunedinPACE between ages 55–60 and 60–65. PC‐Horvath, PC‐Hannum, PC‐PhenoAge, PC‐GrimAge consistently peaked at baseline age 50–55 for the 5‐year heritability change, indicating that for these three clocks, the ages 50–55 and 55–60 represent critical periods for changes in genetic influences.

The longitudinal study on the changes in genetic contributions for each age group was also conducted on the CpGs from four epigenetic clocks, which are documented in Tables S23–S26.

## 4 DISCUSSION

In this twin study, we reported crucial age windows in the variation of genetic factors driving epigenetic clocks and identified the methylation sites which may underlying these changes. First, for all five epigenetic clocks, heritability showed a generally declining trend between the ages of 31 and 70, initially following a relatively flatted trajectory. Subsequently, with the exception of PCGrimAge, the remaining four clocks exhibited a marked decline in heritability: DunedinPACE and PC‐PhenoAge experienced a pronounced decrease between the ages of 55 and 65, while PC‐Horvath and PC‐Hannum showed a similar decline between the ages of 60 and 70. Cluster analysis identified 41, 26, 4, and 36 CpG sites that were potentially responsible for the changes in the heritability of DunedinPACE, PC‐Horvath, PC‐Hannum, and PC‐PhenoAge, respectively. In the longitudinal analysis, we observed varying degrees of decline in the genetic components of the five clocks when compared to baseline. However, corresponding CpGs from these clocks exhibited diverse trends of decrease or increase. DunedinPACE exhibited a peak in the five‐year change in heritability from age 55–60 to 60–65, while PC‐Horvath, PC‐Hannum, PC‐PhenoAge, and PC‐GrimAge consistently reached peaks from age 50–55 to 55–60.

### 4.1 Changes in the heritability of the epigenetic clocks and their CpGs with aging

This study reported the general heritability estimates for five epigenetic clocks, ranging from 0.58 to 0.72, and it was observed that heritability of all five clocks generally exhibited a declining trend from ages 31 to 70. The relevant twin research concerning methylation clocks was limited. One study comprising 31 female twin pairs estimated the heritability of the Horvath clock at 39% (Horvath, 2013). Another study, which included a combined sample of 104 Swedish and Danish twin pairs, conducted follow‐up assessments on the same subjects to evaluate the changes in heritability of the Horvath clock, revealing a heritability of 55% at baseline (age 69) and 51% at follow‐up (age 79) (Jylhava et al., 2019). The other investigation, which involved 1249 twin individuals aged between 20 and 72 years, also examined the heritability of the Horvath clock and found that the influence of genetic factors on the clock was lower for older participants (aged 55 to 70) compared to younger individuals (aged 20 to 25), with heritability estimates of 53% and 74% respectively (Sillanpaa et al., 2019). There is one additional study, based on 285 twin pairs aged 21–25 and 235 twin pairs aged 55–74 and suggested that methylation age displays high heritability in both younger and older twins (60%) (Kankaanpaa et al., 2021).

Our research contributes new evidence illustrating the trends of variations in the heritability of epigenetic clocks with advancing age. DunedinPACE and PC‐PhenoAge exhibited a clear reduction in heritability between ages 55 and 65, whereas PC‐Horvath and PC‐Hannum demonstrated a comparable decrease between ages 60 and 70. In contrast, the heritability for PC‐GrimAge was relatively constant with aging. Differences in the training phenotypes may account for the differences in these trajectories. Horvath and Hannum clocks belong to the first‐generation epigenetic clocks, while PhenoAge, GrimAge and DunedinPACE represent the second and third generations, respectively (Duan et al., 2022; Hannum et al., 2013; Horvath, 2013; Lu et al., 2019). Chronological age was utilized as the training phenotype for establishing the first‐generation epigenetic clocks with the objective of predicting it. Horvath and Hannum clocks incorporate 353 and 71 chronological age‐related CpGs, respectively, and both exhibit correlations of up to 0.96 with chronological age, which may explain the similar trajectory of heritability changes with age observed in these two clocks. Additionally, among the CpGs that identified in our study to potentially affect the change trends of heritability for these two clocks, cg09809672 (annotated from *EDARADD*) were found for both clocks, which have been verified to have an independent crucial role on age prediction (Pan et al., 2020).

One plausible reason for this clear decline of PC‐Horvath and PC‐Hannum could be that study participants aged 60–70 significantly differ from the average age of the training populations, which were 36 and 40 years old for Horvath and Hannum clocks, respectively. The original study of the Horvath clock stated that the correlation of the epigenetic clock with chronological age is determined by the standard deviation of age, suggesting that there may be biases when using this epigenetic clock in younger and older populations (Horvath, 2013). Another possible explanation for the drop in heritability is that the influence of genetic factors truly has changed. During the process of aging, methylation patterns have been demonstrated to exhibit a non‐linear trend of change, but the overall landscape is still unclear (Okada et al., 2023). Also, genetic factors that influencing DNAm process exhibit significant differences in olderly populations compared to those in youth (Shah et al., 2014). Further insights into these variations of genetic factors implicated in the aging process may be obtained through an understanding of the CpG sites derived from the clocks and their corresponding genes. In our analysis on methylation clocks sites, we discovered that the genetic contributions change trajectories for 26/4 CpGs in total were aligned with that of PC‐Horvath/PC‐Hannum clocks, which may potentially play a significant role on the trend of change in heritability for both clocks. Although there is currently no evidence indicating changes in the genetic contributions of these GpGs between the ages of 61 to 70, many genes that annotated from these CpGs have been confirmed to be involved in the pathogenesis and progression of conditions including telomere shortening (Starr et al., 2008), cardiovascular disease (Wu et al., 2024), muscle weakness (Wang et al., 2024), hematopoietic dysfunction (Prall et al., 2007; Williams et al., 2023), during the aging process. These age‐associated diseases that are common in older age groups may also be indicative of significant changes in genetic influences, which may affect methylation in turn.

The PhenoAge clock, developed by Levine et al., utilized DNA methylation data along with biomarkers that related to mortality risk, including chronological age, albumin levels, creatinine, glucose, and C‐reactive protein, was consisted of 513 CpG sites (Levine et al., 2018). Compared to the Horvath clock, the PhenoAge clock demonstrates an evident advantage in predicting mortality risk. For each increase year in PhenoAge clock is associated with a 4.5% increase in the risk of all‐cause mortality, which has presented great potential in research on cancer and cardiovascular diseases (Dugué et al., 2018; Levine et al., 2018; Oblak et al., 2021). DunedinPACE employed longitudinal changes in biomarkers as the training phenotypes, can give measurements on the speed of aging and reflect a range of physiological changes in humans associated with aging, as well as the mortality rate (Belsky et al., 2020, 2022). In this study, the heritability of epigenetic age for both PC‐PhenoAge and DunedinPACE showed a marked decline between the ages of 55 and 65, while remaining relatively stable in the age ranges before and after this period. Additionally, the trends for both clocks were relatively consistent.

On the other hand, the heritability of PC‐GrimAge remained highly stable between the ages of 31 and 70. The GrimAge clock, developed by Lu et al., is composed of 1030 CpG sites, trained using 7 plasma proteins, chronological age, and smoking information (Lu et al., 2019). Contrast to PhenoAge, which is closely associated with a variety of diseases, GrimAge's training phenotype incorporates sex and pack‐years of smoking, factors more related to lifestyle, potentially explaining its relatively stable heritability. Additionally, PC‐GrimAge is the epigenetic clock most strongly correlated with chronological age in this population, which may also contribute to its stable heritability.

Aging is certainly a complex process, involving a host of changes at the cellular and molecular levels, which include chronic, progressive inflammatory response, stem cell senescence, telomere shortening, and changes in gene expression (Jones et al., 2015). Many of these mechanisms were characterized by persistent and progressive changes (Chen & Kerr, 2019; Navarro‐Pando et al., 2021; Robin et al., 2020).

A total of 36 and 41 CpGs were identified to have similar decreasing trajectories with PC‐PhenoAge and DunedinPACE, respectively. Among these sites, several have been found to be involved in the pathological processes of various age‐related diseases such as cardiometabolic diseases, type 2 diabetes, Alzheimer's disease, and coronary heart disease (Ma et al., 2020; Neumann et al., 2022; Nikpay, 2023; Ochoa‐Rosales et al., 2020). There is evidence from research indicating that during aging, methylation levels are widely regulated by genes (Koczor et al., 2016). Future research investigating the role of methylation on aging process is required to further explore these CpGs and the genes they are located in.

### 4.2 Longitudinal changes in the genetic contributions of the epigenetic clocks and their CpGs with aging

In the longitudinal analysis, similar to the cross‐sectional study findings, there was an observed decline in the genetic components of all five clocks from baseline to follow‐up, with the decrease being statistically significant in the four PC clocks. However, the CpGs across all five clocks demonstrated varying trends of decrease or increase. By assessing the longitudinal heritability changes with age groups, we revealed the age 55–60 to be a peak in the 5‐year change in genetic contributions for DunedinPACE, while age 50–55 was for PC‐Horvath, PC‐Hannum, PC‐PhenoAge, and PC‐GrimAge, suggesting the genetic effects may undergo significant changes between the ages of 50–65. To the best of our knowledge, there is currently no longitudinal research investigating the heritability changes of the epigenetic clock and their CpGs. Age 60 is well‐accepted as a critical aging milestone. Around the age of 60, the pace of human aging accelerates, and various physiological functions proceed to an advance age stage (Han, 2024; Seo & Ryu, 2022). Evidence has been presented that during aging process, the monotonous demethylation pattern changes around the age of 60, which has been identified as a stable nonlinear pattern (Okada et al., 2023). At the age of 55, women are influenced by menopausal hormones and endocrinologic changes, which can lead to significant changes in genomics, transcriptomics, proteomics, metabolomics (Li et al., 2023). It has also been established that individuals aged 55 and above are at risk for the development of various chronic diseases like stroke and cancer (Kim et al., 2021; Zhou et al., 2022). In this study, based on a twin study design, we suggested that age 55–60 and 60–65 may be pivotal for alterations in the genetic contributions to the DunedinPACE and the PC‐Horvath/PC‐Hannum/PC‐PhenoAge/PC‐GrimAge clocks, respectively. Due to the different methodologies implemented in the establishment of these clocks, they reflect different aspects of the aging process. Furthermore, it is noteworthy that the genetic variance components of certain CpGs from these clocks exhibited an increasing trend over time in this study. This may suggest that new genetic effects emerge as age increases. Among these, many CpGs have been demonstrated to associate with age‐related diseases. For instance, cg00574958 was related to type 2 diabetes and obesity (Cardona et al., 2019; Karlsson et al., 2020) and cg02650017 was for dyslipidemia (Sayols‐Baixeras et al., 2018). It is well known that aging process is accompanied by progressively changes in the expression of multiple genes and an increase of genomic methylation (Rebbani et al., 2015). Changes in genetic factors can lead to alterations in the pace of aging, in turn, accelerated aging may also induce changes in gene expression through regulatory mechanisms such as methylation, potentially resulting in a series of intestinal pathological alteration like inflammatory responses (Reynolds et al., 2014). Current research has identified several thousand of CpG sites that potentially causal for age‐related traits by conducting Mendelian randomization analysis across the epigenomic landscape (Ying et al., 2024). Implications for future research and trials could target on investigating the effects of these CpGs specifically in the olderly population, examining the plausibility of modulating the genetic impact influencing these sites to alter their methylation patterns and reduce the adverse outcomes.

### 4.3 Strengths and limitations

This study has several strengths. First, to the best of our knowledge, this is the first study to analyze the trends of heritability changes with aging for epigenetic clocks across a broad range of ages. Previous research on metabolomics, proteomics, and genomics has explored critical periods of aging and related changes, and numerous related biomarkers have been proposed. However, no study has yet quantitatively analyzed the variations in the genetic contributions by age to epigenetic clocks. The twin population offers a unique advantage for this topic as twin designs allow for the assessment of the effects of genetic variance. Second, based on the longitudinal data, our study is the first to evaluate the 5‐year longitudinal change in heritability of these clocks, indicating significant age periods where genetic influences have longitudinal alterations, thus providing further insights into the genetic contributions behind the dynamics of aging. Third, our research conducted a comprehensive assessment of the trend in the changes in heritability with aging of these clocks' sites, both longitudinally and cross‐sectionally, identifying dozens of CpGs that were potentially related to the changes in genetic influence on these aging biomarkers.

The study also has several limitations. Firstly, due to the differences of ethnicity in populations that used to establish these epigenetic clocks, there may be limitations on the generalizability of the findings from this study. It has been demonstrated that methylation clocks may exhibit certain racial disparities (Akhabir et al., 2022). Future research should further investigate the genetic influences on these clocks in other populations and ethnicities. Secondly, the sample size, especially for longitudinal analysis, is relatively small, which may introduce certain boundary effects. Gender may have an impact on methylation patterns and genetic effects, yet stratification by gender was not feasible in this study due to the limited study population. Larger sample sizes will be needed in the future to study the influences on genetic factors of epigenetic clocks varied with age. Moreover, due to the rarity of twins as a research resource, collecting twin pairs presents significant challenges, leading to the majority in this cohort are recruited voluntarily. Consequently, the study population from CNTR may be subject to selection bias and may not fully represent the entire twin population in China. Thirdly, this study can only provide a quantitative measure of the genetic influence, while the specific mechanisms of its impact remain unclear and require further exploration.

## 5 CONCLUSIONS

In summary, we assessed the changes in the heritability of epigenetic clock indicators with aging. We detected that the overall heritability of all five epigenetic clocks declined between the ages of 31 and 70, begin with a relatively stable period. Subsequently, except for PC‐GrimAge, the other four clocks demonstrated an evident reduction in heritability: DunedinPACE and PC‐PhenoAge experienced a marked decline between the ages of 55 and 65, while PC‐Horvath and PC‐Hannum showed a similar decrease between 60 and 70 years of age. Similar analyses were conducted on the CpGs from these clocks, and cluster analysis revealed 41/26/4/36 for DunedinPACE/PC‐Horvath/PC‐Hannum/PC‐PhenoAge that may be implicated in heritability changes. Longitudinal analyses disclosed that genetic contributions were declined for all five clocks from baseline to follow‐up, with the decrease being statistically significant in the four PC‐clocks. For different age groups, DunedinPACE peaked during the 5‐year change in genetic contributions at age 55–60, while for PC‐Horvath, PC‐Hannum, PC‐PhenoAge, and PC‐GrimAge, the peak was observed at age 50–55. These findings offer novel insights into the understanding of the role of genetic variations on the process of aging.

## AUTHOR CONTRIBUTIONS

**Wenjing Gao**: Conceptualization, methodology, writing—review and editing; **Ruqin Gao**, **Min Yu**, **Jinyi Zhou**, **Xianping Wu**, **Yu Liu, and Shengli Yin**: Investigation. **Hui Cao**, **Weihua Cao**, **Jun Lv**, **Canqing Yu**, **Tao Huang**, **Dianjianyi Sun**, **Chunxiao Liao**, **Yuanjie Pang** and **Runhua Hu**: Writing—review and editing. **Liming Li**: Supervision, project administration, funding acquisition. **Xuanming Hong**: Software, writing—original draft.

## CONFLICT OF INTEREST STATEMENT

The authors declare that they have no competing interests.

## Supporting information


**Data S1:** Supporting information.

**Data S2:** Supporting information.

## Data Availability

The datasets analyzed in this study are not publicly available due to the informed consent involved in this study stating that the data of the participants could not be disclosed to a third party. But the data that support the findings of this study are available on request from the corresponding author Prof. Wenjing Gao.
