# Supplementary material for: Trends of genetic contributions on epigenetic clocks and related methylation sites with aging: A population‐based adult twin study
Source: Aging Cell. 2024 Nov 14;24(3):e14403. doi: 10.1111/acel.14403 (PMC11896513; doi:10.1111/acel.14403)
Supplement: Supplementary file 1 — Data S1: Supporting information. [file ACEL-24-e14403-s001.docx]

**Supplementary Materials**

**Supplementary Figures**


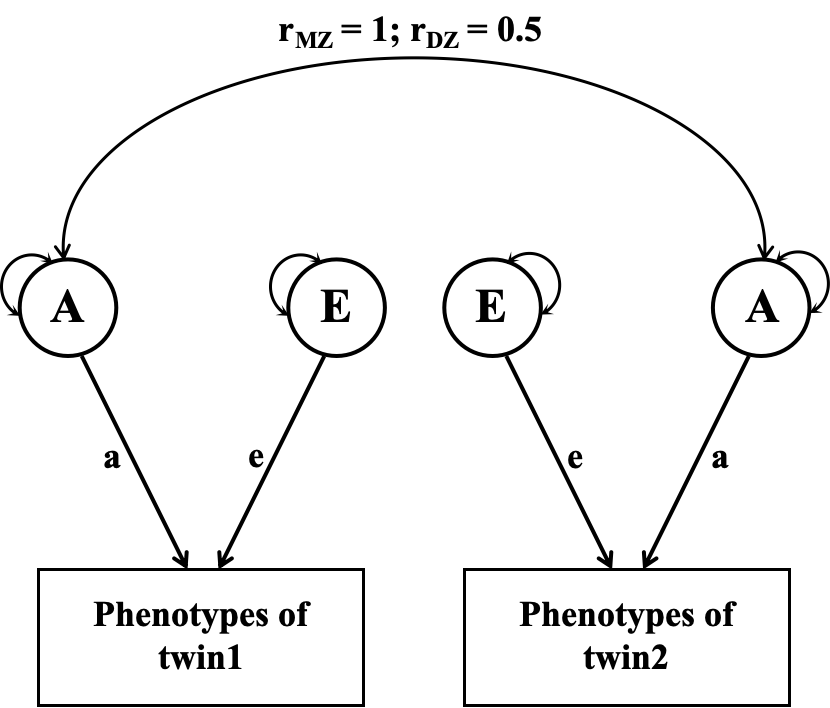


**Supplementary Figure 1 Diagram for the univariate structural equation model consisting of A and E components**

The phenotypes for a twin pair are depicted in squares, while latent factors are in circles. The correlations of additive genetic variance (A) are 1 in MZ twins and 0.5 in DZ twins. The correlations of Unique environmental variance (E) are consistently 0.


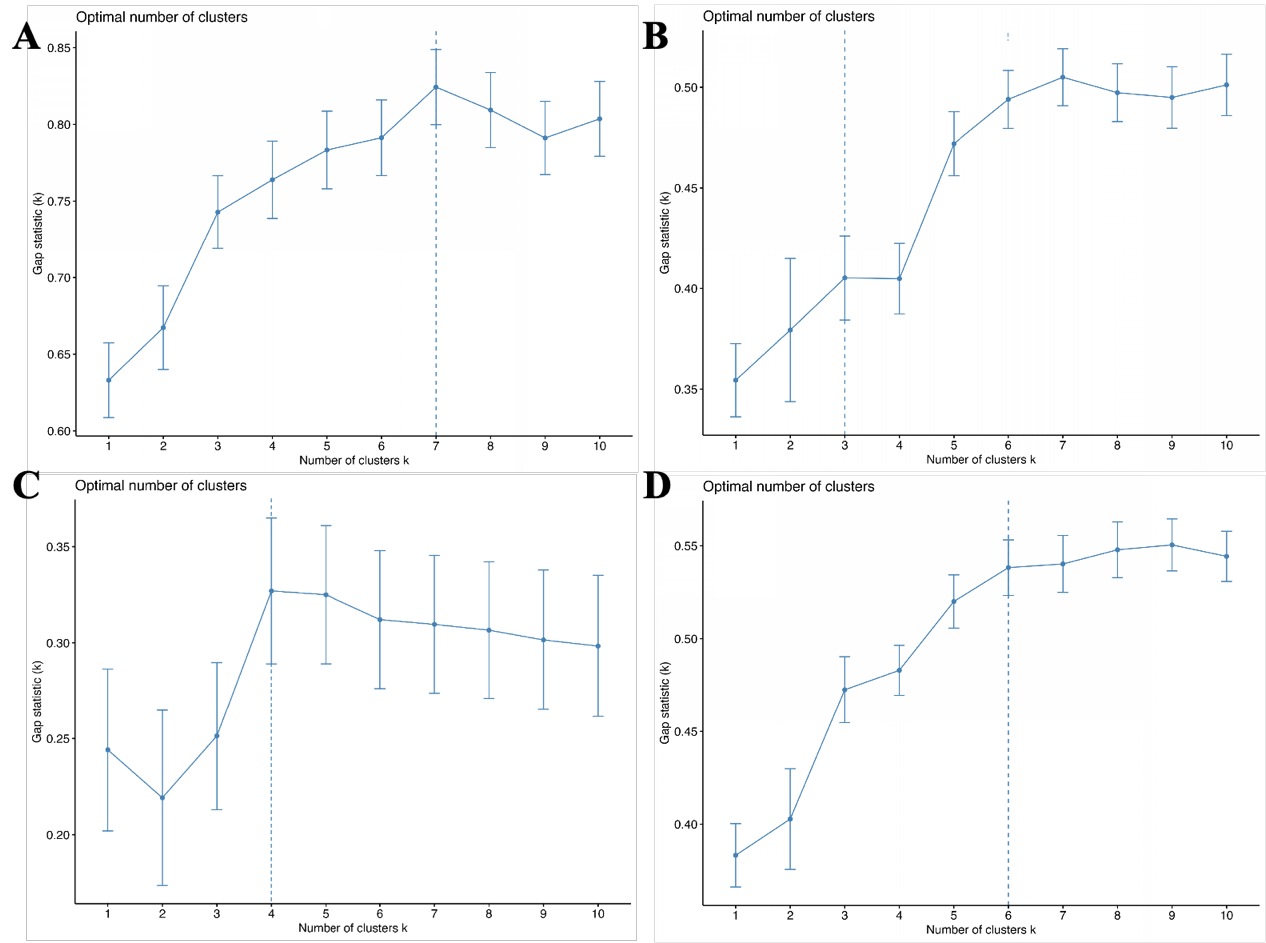


**Supplementary Figure 2 Gap statistic curves for clustering analysis on the trajectory of heritability changes with age of epigenetic clocks and their CpGs**

Gap statistic curves for (A) DunedinPACE and its CpGs; (B) PC-Horvath and its CpGs; (C) PC-Hannum and its CpGs; (D) PC-PhenoAge and its CpGs
